# 894. Trend of Transmitted Resistance Associated Mutations in People Living with HIV (PLWH) in a Large Southeastern U.S. Ryan White Clinic

**DOI:** 10.1093/ofid/ofab466.1089

**Published:** 2021-12-04

**Authors:** Alisha Kavouklis, Amber F Ladak, Caroline Hamilton, Annastesia Mims, Gina Askar, Cheryl Newman

**Affiliations:** 1 Augusta University, Waynesboro, Georgia; 2 Augusta University/Medical College of Georgia, Augusta, Georgia

## Abstract

**Background:**

Department of Health and Human Services (DHHS) guidelines recommend integrase strand transfer inhibitors (INSTIs) as the backbone of preferred initial antiretroviral (ART) regimens (1). Baseline mutation rates for the INSTI class is 0.8% compared with an overall rate of 19% for all ART classes, based on Centers for Disease Control and Prevention (CDC) U.S. data from 2013-16 (2). First-generation INSTIs (raltegravir and elvitegravir) have a lower genetic barrier to resistance compared with newer, second generation INSTIs (bictegravir and dolutegravir) (3, 4). DHHS guidelines do not currently recommend routine HIV genotypic resistance testing to INSTIs prior to ART initiation (1). Our study seeks to determine the current prevalence of transmitted INSTI and overall resistance in a large southeastern U.S. Ryan White clinic.

**Methods:**

This was a single-center, retrospective analysis of treatment naïve PLWH presenting for care from January 1, 2017 to December 31, 2020. Of these, 164 had a baseline genotype performed by one of two commercially available assays – Vela Genomics or ViroSeq. Subsequent interpretations were based on Stanford HIV Drug Resistance Database.

**Results:**

65 patients (39.6%) had at least one transmitted resistance associated mutation (RAMs). Of these, 24 (36.9%) had an INSTI RAM. Baseline PI, NRTI, and NNRTI RAMs declined during the four-year interval (2017-2020), while the rate of INSTI RAMs increased from 11.1% to 19%; all conferred resistance to the first generation INSTIs with one also conferring resistance to second generation INSTIs.

INSTI Resistance Associated Mutation Prevalence 2017-2020

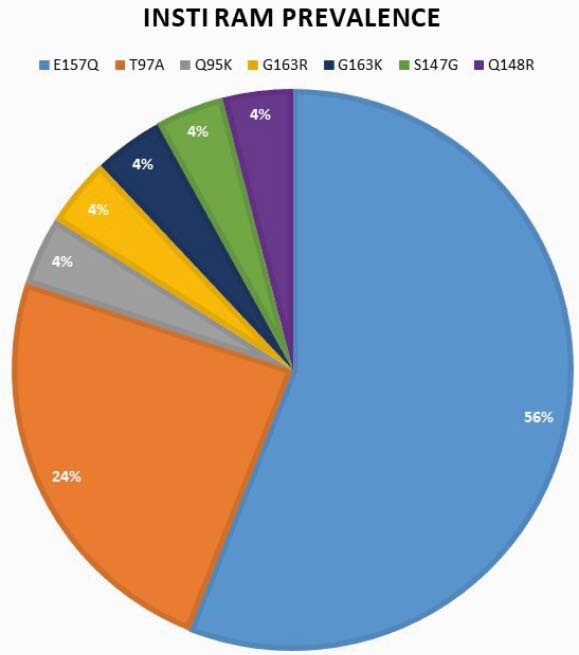

Frequency of Antiretroviral Therapy Class Mutations Per Year

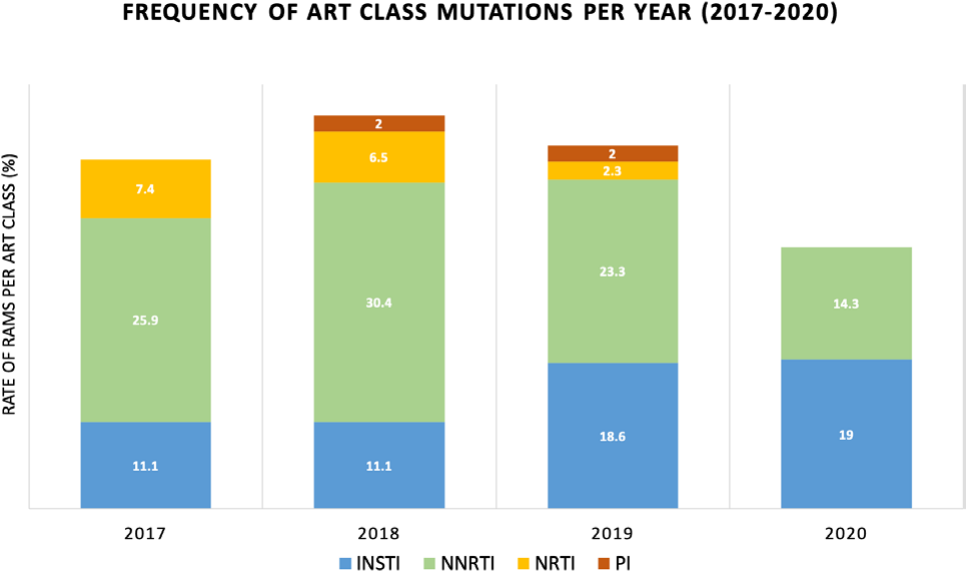

Trend of INSTI Mutations and Resistance Associated Mutations 2017-2020

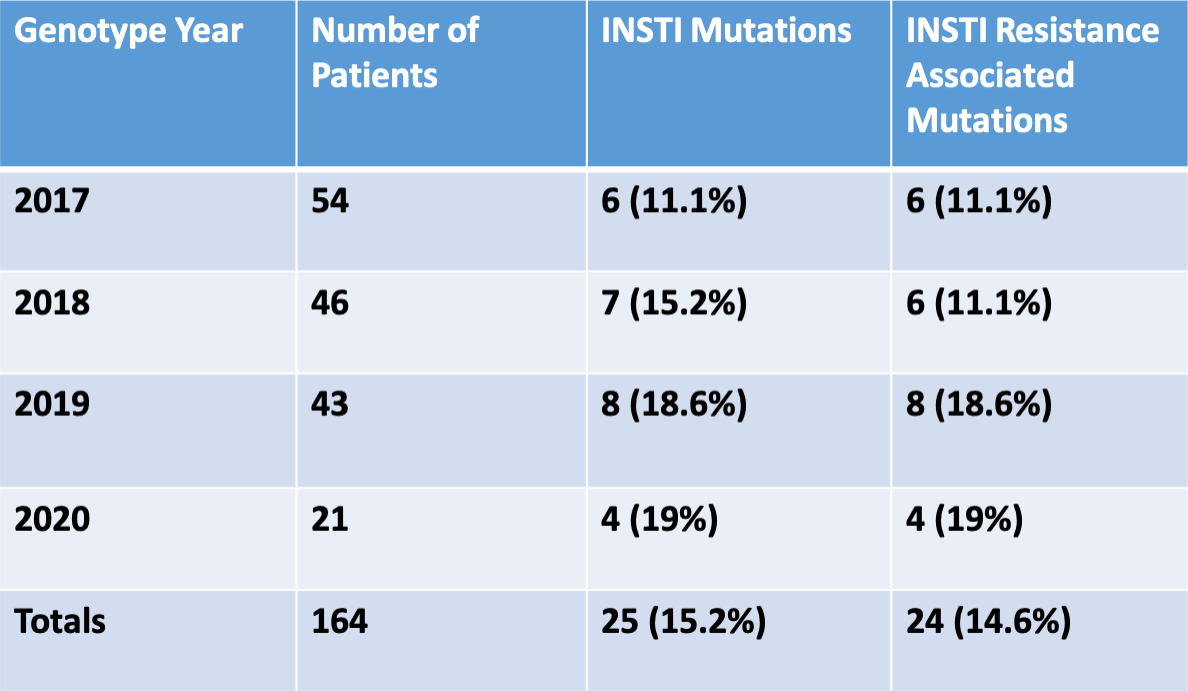

**Conclusion:**

Unlike the CDC data which showed the overall prevalence of INSTI RAM transmission rates during 2013-2016 to be 0.8%, our data suggests a higher rate of INSTI RAMs (14.6%) with overall ART RAM transmission of 39.6%. This increase in baseline resistance to the INSTI class, which occurred over time, mimics the historical development of RAMs seen in the earlier ART classes. Though suboptimal adherence in the population promotes development of RAMs, increased frequency of INSTI RAMs may be due to a lower barrier to resistance of first generation INSTIs. Should our observed trend continue, routine baseline INSTI resistance testing may need to be considered prior to ART initiation.

**Disclosures:**

**Cheryl Newman, MD**, **Gilead** (Scientific Research Study Investigator)**GSK/ViiV** (Scientific Research Study Investigator, Advisor or Review Panel member, Speaker’s Bureau)**Janssen** (Scientific Research Study Investigator)**Merck** (Scientific Research Study Investigator)

